# Supplementary material for: Fused-Deposition Modeling 3D Printing of Short-Cut Carbon-Fiber-Reinforced PA6 Composites for Strengthening, Toughening, and Light Weighting
Source: Polymers (Basel). 2023 Sep 11;15(18):3722. doi: 10.3390/polym15183722 (PMC10534845; doi:10.3390/polym15183722)
Supplement: Supplementary file 1 [file polymers-15-03722-s001.zip › polymers-2599636-supplementary.pdf]

Supplementary Materials

Fused-Deposition Modeling 3D Printing of Short-Cut Carbon -Fiber-Reinforced PA6 Composites for Strengthening, Toughening, and Light Weighting

Bin Sun [1,2,3], Suhail Mubarak[4], Guocun Zhang[5], Kangming Peng[1,6], Xueling Hu[1,7], Qia Zhang[8], Linxin Wu [1,2*] and Jianlei Wang [1,2,3*]

1 CAS Key Laboratory of Design and Assembly of Functional Nanostructures, Fujian Key Laboratory of Nanomaterials, Fujian Institute of Research on the Structure of Matter, Chinese Academy of Sciences, Fuzhou 350002, China
2 University of Chinese Academy of Sciences, Beijing 100049, China
3 CAS Haixi Industrial Technology Innovation Center in Beilun, Ningbo 315830, China
4 State Key Laboratory of Fluid Power & Mechatronic System, Key Laboratory of Soft Machines and Smart Devices of Zhejiang Province, Center for X-Mechanics, and Department of Engineering Mechanics, Zhejiang University, Hangzhou, 310027 P. R. China
5 School of Automotive Engineering, Dalian University of Technology, Dalian, 116024, China
6 College of Chemistry and Materials Science, Fujian Normal University, Fuzhou 350007, China
7 College of Chemistry, Fuzhou University, Fuzhou 350116, China
8 Chunhui Technology Group Co., Ltd, Fuzhou 350019, China
* Correspondence: lxwu@fjirsm.ac.cn (L.W.); jlwang@fjirsm.ac.cn (J.W.)

Figures

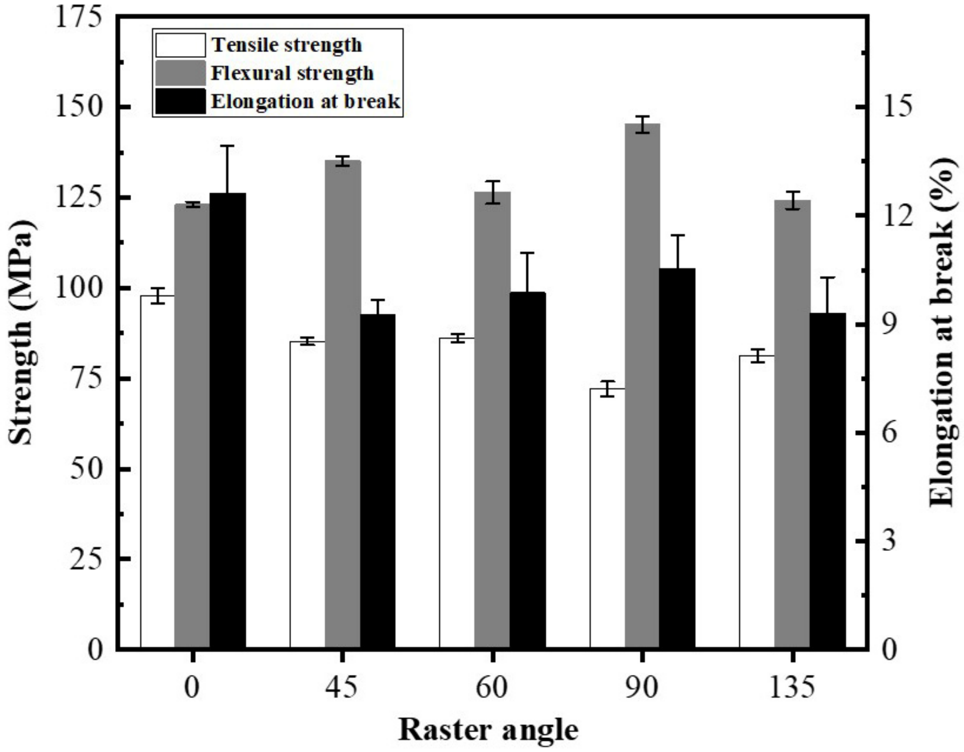

Figure S1: Mechanical properties of 3D-printed composites with different raster angles

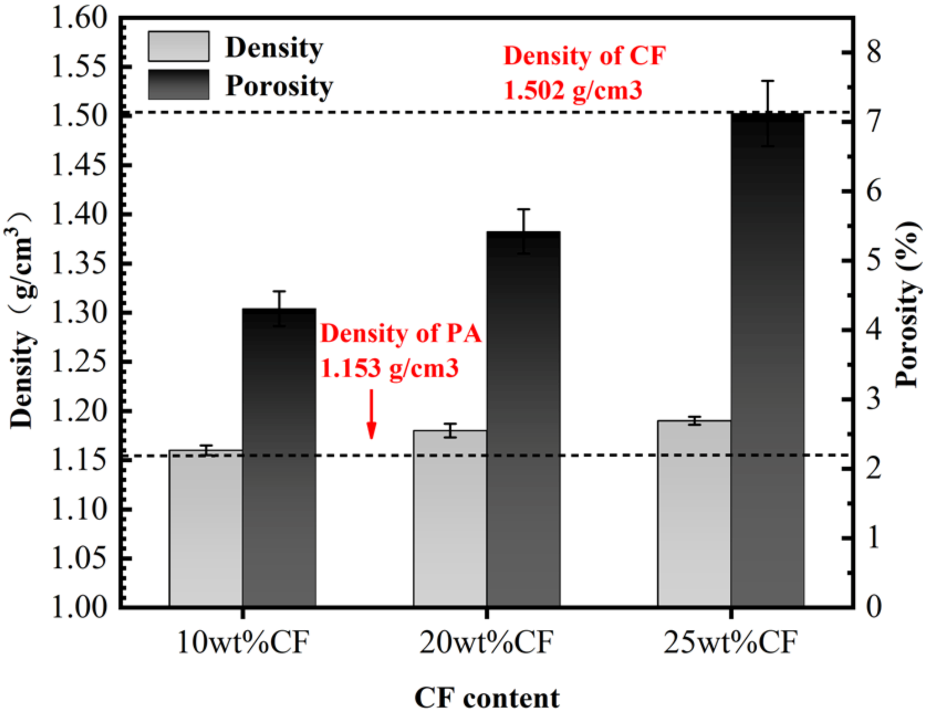

Figure S2: Density and porosity of 3D printed composites with different fiber contents

Tables

Table S1. Results of Three-Factor Orthogonal Experiment on Print Parameter Impact on Mechanical Performance

Factor	Tensile strength μ_i	Tensile strength σ_i^2	Flexural strength μ_i	Flexural strength σ_i^2	Impact strength μ_i	Impact strength σ_i^2
Fiber contents 10wt%/20wt%/25wt%	74.13	284.74	118.90	57.25	16.97	26.55
	66.88	137.62	129.83	150.27	15.80	54.45
	60.60	164.58	118.97	51.23	17.43	29.56
$\overline{\mu_i}$ $\overline{\sigma_i^2}$	67.20	195.65	122.57	86.25	16.73	36.85
Raster angle 0°/45°or135°/90°	54.78	22.49	112.48	14.53	11.30	4.99
	61.10	8.84	121.18	48.11	14.13	4.55
	85.64	45.55	131.93	80.59	24.08	3.51
$\overline{\mu_i}$ $\overline{\sigma_i^2}$	67.17	25.62	121.86	47.74	16.50	4.35
Build-up temperature 60°C/90°C	72.70	73.96	129.65	141.80	20.60	11.56
	69.40	68.89	129.30	78.41	20.07	30.25
	75.25	101.03	126.4	89.43	21.20	15.21
$\overline{\mu_i}$ $\overline{\sigma_i^2}$	72.45	81.29	128.45	103.21	20.80	19.01

Based on the results shown in **Table S1**, a three-factor orthogonal experiment was conducted on the mechanical performance data in **Table 2**. The results indicate that the parameters of 3D printing (fiber content, raster angle, and build-up temperature) have different degrees of influence on the mechanical performance of the composite material. According to the average variance, the ranking for tensile strength is: fiber content > build-up temperature > raster angle. For bending strength, the ranking is: build-up temperature > fiber content > raster angle. For impact strength, the ranking is: fiber content > build-up temperature > raster angle.
